# Prevalence and genotypic relatedness of carbapenem resistance among multidrug-resistant *P. aeruginosa* in tertiary hospitals across Thailand

**DOI:** 10.1186/1476-0711-11-25

**Published:** 2012-09-13

**Authors:** Piyatip Khuntayaporn, Preecha Montakantikul, Piroon Mootsikapun, Visanu Thamlikitkul, Mullika Traidej Chomnawang

**Affiliations:** 1 Department of Microbiology, Faculty of Pharmacy, Mahidol University, 447 Sri Ayudthaya Road, Rachathevi, Bangkok, 10400, Thailand; 2 Department of Pharmacy, Faculty of Pharmacy, Mahidol University, Bangkok, Thailand; 3 Department of Medicine, Faculty of Medicine, Khon Kaen University, Khon Kaen, Thailand; 4 Department of Medicine, Faculty of Medicine Siriraj Hospital, Mahidol University, Bangkok, Thailand

**Keywords:** Antimicrobial susceptibility, Pulsed-field gel electrophoresis, Carbapenem resistance, Multidrug resistance, *Pseudomonas aeruginosa*, Epidemiology

## Abstract

**Background:**

Increased infection caused by multidrug resistant (MDR) *Pseudomonas aeruginosa* has raised awareness of the resistance situation worldwide. Carbapenem resistance among MDR (CR-MDR) *P. aeruginosa* has become a serious life-threatening problem due to the limited therapeutic options. Therefore, the objectives of this study were to determine the prevalence, the antibiotic susceptibility patterns and the relatedness of CR-MDR *P. aeruginosa* in tertiary hospitals across Thailand.

**Methods:**

MDR *P. aeruginosa* from eight tertiary hospitals across Thailand were collected from 2007–2009. Susceptibility of *P. aeruginosa* clinical isolates was determined according to the Clinical and Laboratory Standards Institute guideline. Selected CR-MDR *P. aeruginosa* isolates were genetically analyzed by pulsed-field gel electrophoresis.

**Results:**

About 261 clinical isolates were identified as MDR *P. aeruginosa* and approximately 71.65% were found to be CR-MDR *P. aeruginosa.* The result showed that the meropenem resistance rate was the highest reaching over 50% in every hospitals. Additionally, the type of hospitals was a major factor affecting the resistance rate, as demonstrated by significantly higher CR-MDR rates among university and regional hospitals. The fingerprinting map identified 107 clones with at least 95% similarity. Only 4 clones were detected in more than one hospital.

**Conclusions:**

Although the antibiotic resistance rate was high, the spreading of CR-MDR was found locally. Specific strains of CR-MDR did not commonly spread from one hospital to another. Importantly, clonal dissemination ratio indicated limited intra-hospital transmission in Thailand.

## Introduction

Overuse of antibiotics in a hospital can cause a selective pressure on microorganisms, which in turn, can enhance the antimicrobial resistance in bacteria. Inappropriate use of antibiotics has been reported to be involved in increasing the antibiotic resistance [1,2]. This circumstance becomes a major concern in Thailand, especially in university hospitals [3].

Among hospital-acquired microorganisms, *Pseudomonas aeruginosa*, a non-fermentative, gram-negative bacterium, is one of the most common causative agents in nosocomial infections. According to the NNISS report (National Nosocomial Infections Surveillance System in United States) and INICC (International Nosocomial Infection Control Consortium), *P. aeruginosa* is the most common pathogen found in intensive care units from the respiratory tract and central line-associated primary bloodstream infections [4-6]. Moreover, the report from the SENTRY program in Latin American during 1997–2000 showed that *P. aeruginosa* was the most commonly isolated pathogen from hospitalized pneumonia patients and had high resistance rates for most of tested antimicrobials [7]. In Thailand, like other parts of the world, *P. aeruginosa* has been shown to have the highest prevalence rate along with its intrinsic antibiotic resistance mechanisms [8,9]. Infections caused by multidrug resistant (MDR) *P. aeruginosa* can lead to serious outcomes such as amputation or in the worst case, death [10]. The mortality rate of MDR *P. aeruginosa* infections was significantly higher than infections caused by susceptible *P. aeruginosa*[11]. In the US surveillance study, MDR *P. aeruginosa* rate was found to increase from 4% to 14% over the period of 1993 to 2002 [4].

Carbapenem, a member of the β-lactam family, has a broad spectrum of activity and is stable to most β-lactamases. These properties make carbapenem to be important therapeutic options for treating serious infections involving resistant strains of Enterobacteriaceae, anaerobes, *P. aeruginosa*, and *Acinetobacter* spp. [12,13]. Carbapenem has always been chosen as the first option for initial empirical treatment in many severe infections, e.g. nosocomial pneumonia, and chronic MDR pseudomonal infections [12,14]. Although carbapenem is a very powerful antibiotics, the resistance rate is still increasing [15].

Although carbapenem resistance (CR) has been widely studied, there is limited information on the CR rates among MDR (CR-MDR) bacterial pathogens. These situations raised the question, “How many *P. aeruginosa* isolates in Thailand are CR-MDR?” Therefore, the objectives of this study were to determine (i) the prevalence of CR-MDR *P. aeruginosa* in Thailand, (ii) the antibiotic susceptibility patterns of CR-MDR *P. aeruginosa*, and (iii) the relatedness of CR-MDR *P. aeruginosa* clinical isolates across tertiary hospitals in Thailand. This study would provide the first update report on an epidemiology of CR-MDR *P. aeruginosa* in Thailand.

## Materials and methods

### Bacterial collection

*P. aeruginosa* clinical isolates were collected from eight hospitals from 2007 to 2009 within five regions of Thailand. The study was complied with International Guidelines for Human Research Protection and was approved by Mahidol University Institutional Review Board (MU-IRB) under the certificate of approval No. MU-IRB 2011/025.0102. All participated hospitals are tertiary hospitals. Four hospitals are regional hospitals and the other four hospitals are university hospitals. Regional hospitals located in province centers have a capacity for at least 500 beds, while university hospitals are teaching schools which also provide postgraduate and specialist programs. Both hospitals have a comprehensive set of specialists and have been transferred patients from primary and secondary hospitals in their regions. However, rare condition treatments or patients with high complexity mostly have been transferred to university hospitals. Bacterial clinical isolates were identified and performed susceptibility test by the hospital’s laboratory technicians using their standard hospital procedures. Multidrug resistance criteria in this study were defined as non-susceptible to at least 3 of 5 drug groups, including the anti-pseudomonal penicillins, cephalosporins, aminoglycosides, fluoroquinolones and carbapenem [16]. Bacterial strains were cultured in triple sugar iron agar (BD, Sparks, MD, USA) and stored at 4°C before being submitted to the research laboratory. All bacteria clinical isolates were enriched before storage at −80°C. Cetrimide agar (BD, Sparks, MD, USA) supplement with 10% glycerol was used as the selective minimal medium to confirm isolates as *P. aeruginosa*.

### Antibiotic susceptibility test

The susceptibility of *P. aeruginosa* clinical isolates was confirmed in the research laboratory by the disc diffusion method according to the Clinical and Laboratory Standards Institute (CLSI) guidelines [17]. All antibiotic discs in this study included piperacillin (PIP), ceftazidime (CTZ), ciprofloxacin (CIP), gentamicin (CN), imipenem (IMP), meropenem (MEM), and doripenem (DOR) were purchased from Oxoid (Hants, UK). Antibiotic discs were placed onto the inoculated Mueller Hinton agar (BD). After 37°C incubation overnight, inhibition zones were measured and compared to CLSI guidelines [18]. Imipenem and meropenem zone diameter breakpoints were applied to doripenem since the CLSI official zone diameter breakpoints for doripenem were unavailable. Carbapenem resistance was defined by being non-susceptible to at least 1 of the 3 carbapenem tested. Among MDR *P. aeruginosa* clinical isolates, those that were also CR were selected for study.

### Genotyping by pulsed-field Gel electrophoresis

All selected CR-MDR *P. aeruginosa* clinical isolates were analyzed by pulsed-field gel electrophoresis (PFGE) using CHEF Mapper XA system (Bio-Rad, Hercules, CA, USA). PFGE plugs were prepared according to Romling *et al*. with some modifications [19]. The genotyping patterns were confirmed for the relatedness of bacterial isolates by Fingerprinting II Informatix^TM^ software version 3.0 (Bio-Rad). A dendrogram was generated by the unweighted-pair group method. The correlation between band patterns was calculated with dice coefficient. Different clones were considered, if the percentage of similarity was less than 95% [20]. The clonal dissemination ratio in each hospital was calculated by number of CR-MDR *P. aeruginosa* divided by number of selected clones. Selected clones found in more than one hospital were counted in every hospital. The clonal dissemination ratio was indicated intra-hospital transmission.

### Statistical analysis

Statistical analysis was compared between two groups by Student *t*-test. A *p*-value of <0.05 was considered statistically significant. Data were presented as percentages unless otherwise stated.

## Results

### Antimicrobial susceptibility profile

Two-hundred and sixty-one clinical isolates from eight tertiary hospitals across Thailand were selected as MDR *P. aeruginosa*. Antibiotic resistance rates of MDR *P. aeruginosa* in each hospital were shown in Table 1. Ceftazidime demonstrated the highest resistance rate of about 95.79%, followed by ciprofloxacin and gentamicin which were 92.34% and 87.36% resistance, respectively. The carbapenem resistance rate among MDR *P. aeruginosa* isolates was the highest for meropenem (about 65.52%) and the resistance rates for imipenem and doripenem were about 44.44% and 36.02%, respectively. It was noteworthy that meropenem resistance was found at least 50% in all hospitals. Furthermore, resistance rates of carbapenem, unlike resistances for other drugs analyzed, were found to be statistically greater (*p* < 0.05) between a university hospital and a regional hospital.

**Table 1:** **Drug resistance rates of MDR *P. aeruginosa *in participated hospitals **

**Size of Hospital**	**Region of Thailand**	**Hospital**	**IMP (%)**	**MEM (%)**	**DOR (%)**	**PIP (%)**	**CTZ (%)**	**CIP (%)**	**CN (%)**
**University Hospital**	Central	UC	50.00	73.08	19.23	88.46	88.46	88.46	88.46
	Northeast	UNE	59.18	77.55	61.22	67.35	100.00	97.96	71.43
	North	UN	60.98	80.49	56.10	39.02	100.00	100.00	100.00
	South	US	47.62	61.90	42.86	61.90	100.00	95.24	100.00
**Regional Hospital**	Northeast	TNE	41.86	51.16	32.56	76.74	100.00	88.37	95.35
	South	TS	37.50	50.00	20.83	83.33	95.83	91.67	75.00
	East	TE1	20.00	56.67	0.00	100.00	80.00	73.33	76.67
	East	TE2	22.22	62.96	29.63	85.19	96.30	100.00	96.30
**Average**			44.44	65.52	36.02	73.18	95.79	92.34	87.36

PIP, piperacillin; CTZ, ceftazidime; CIP, ciprofloxacin; CN, gentamicin; IMP, imipenem; MEM, meropenem; DOR, doripenem.

Additionally, one-hundred and eighty-seven isolates were determined to be CR-MDR *P. aeruginosa*. An average percentage of CR-MDR compared to MDR *P. aeruginosa* was found to be very high, that is 70.49% with a range of 58.33% to 83.67% (Table 2). The type of a hospital was a major factor affecting the resistance rate as demonstrated by significantly higher CR-MDR rates (*p* < 0.05) among university hospitals and regional hospitals. However, the region where a hospital located was not observed to be a significant determining factor. Moreover, among CR-MDR *P. aeruginosa*, there was no isolates resistant to only doripenem and only one isolate showed double resistance to imipenem and doripenem.

**Table 2:** CR-MDR ratio, clonal dissemination and clone number analyzed by PFGE in participated hospitals

**Size of Hospital**	**Region of Thailand**	**Hospital**	**MDR (N)**	**CR-MDR (N)**	**CR-MDR Ratio (%)**	**PFGE-selected clone (N)**	**Clonal dissemination ratio**	**PFGE clone number (amount of existing clones)**
**University Hospital**	Central	UC	26	21	80.77	17	1.24	18 (4), 8(2), 9, 11, 15, 16, 19, 44, 69, 73, 87, 94, 95, 98, 102, 105, 106
	Northeast	UNE	49	41	83.67	20	2.05	35(9), 45(5), 53(3), 65(3), 84(3), 83(2), 86(2), 89(2),
								13, 41, 46, 50, 51, 54, 56, 62, 85, 88, 99, 107
	North	UN	41	34	82.93	18	1.89	90(6), 92(3), 20(3), 26(3), 28(3), 27(2), 30(2), 68(2),
								12, 14, 21, 23, 24, 25, 29, 31, 91, 93
	South	US	21	15	71.43	11	1.36	10(3), 40(2), 41(2), 2, 17, 33, 42, 43, 52, 66, 70
**Regional Hospital**	Northeast	TNE	43	26	60.47	18	1.44	5(3), 7(3), 3(2), 4(2), 55(2), 79(2), 80(2),
								6, 57, 75, 76, 77, 78, 82, 97, 101, 103, 104
	South	TS	24	14	58.33	10	1.40	32(2), 81(2), 1(2), 2(2), 33, 34, 48, 49, 72, 74
	East	TE1	30	19	63.33	10	1.90	62(4), 63(4), 58(2), 60(2), 64(2), 22, 39, 59, 67, 100
	East	TE2	27	17	62.96	8	2.13	36 (9), 47(2), 37, 38, 41, 61, 71, 96

MDR, multidrug resistant *P. aeruginosa*; CR-MDR, carbapenem resistance among multidrug-resistant *P. aeruginosa*; PFGE, Pulsed-field gel electrophoresis.

* underline indicated the selected clones which were found in more than one hospital.

### Pulsed-field Gel electrophoresis

**Figure 1:**
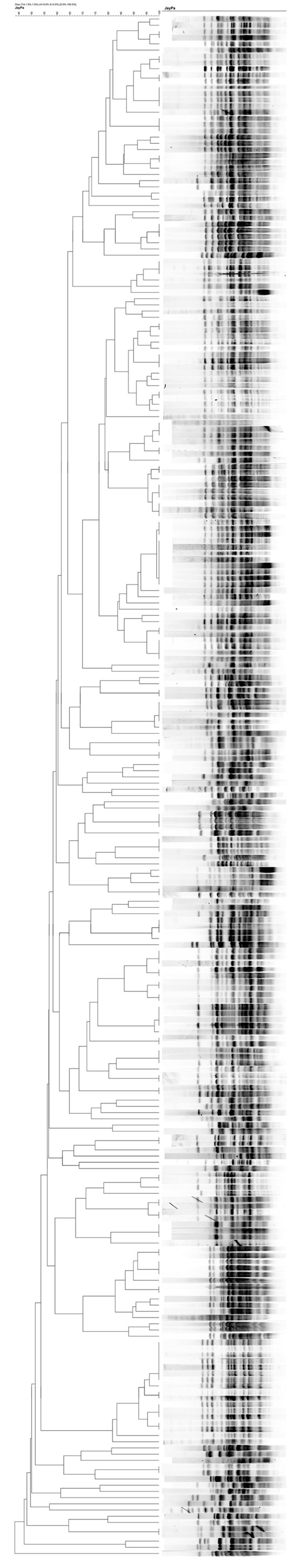
** A fingerprinting map showing the percentage of similarity among 187 strains of CR-MDR *P. aeruginosa. ***

One-hundred and eighty-seven strains of CR-MDR *P. aeruginosa* were analyzed by pulsed-field gel electrophoresis and 107 clones were identified as having 95% similarity (Figure 1). Only 4 clones from 107 clones were detected in more than one hospital (Table 2). Two clones isolated from hospitals of the same region were found to be related. Another clone was found related in hospitals from two separate regions. The last clone was found in three hospitals that were located in three separated regions. Furthermore, the clonal dissemination ratio of higher than 2.0 was found in two hospitals in which the isolates were highly dominated at about 9 isolates per clone. (Table 2)

## Discussions

The multidrug resistance of microorganisms has become the critical problem in nosocomial infections, especially *P. aeruginosa* and *Acinetobacter* spp. Both pathogens have been listed in six famous ESKAPE pathogens (*Enterococcus faeciumStaphylococcus aureusKlebsiella pneumoniaeAcinetobacter* species, *Pseudomonas aeruginosa*, and *Enterobacter* species) and identified as the most emerging threats in this century [21]. Antimicrobial resistance surveillance, has been performed in almost every countries to identify the major problem in nosocomial infections. This study provides the first update data on the genetic relatedness of CR-MDR *P. aeruginosa* clinical isolates from tertiary hospitals across Thailand.

Antimicrobial resistance surveillance studies have been observed in many countries throughout the world. Most of these studies also determined carbapenem resistance rates which described higher resistance rate for imipenem than for meropenem [15,22-24]. However, the Thailand national surveillance during 2000–2005 by Dejsirilert *et al.* was described in a different manner and showed a slightly greater resistance rate for meropenem than for imipenem [25]. Moreover, a recent study which was conducted by Piyakul *et al.* in a tertiary hospital in Thailand indicated that *P. aeruginosa* clinical isolates exhibited a greater meropenem resistance rate than a rate for imipenem [26]. In agreement with the study of Piyakul *et al.*, our data which analyzed MDR isolates of *P. aeruginosa,* showed a greater difference of resistance rates between meropenem and imipenem compared to Piyakul *et al.*[26]. This difference might be caused by the variety of the participated hospitals in the studies. These studies indicated that carbapenem usage in Thailand should be considered when drug susceptibility profile was unavailable.

Although the carbapenem resistance rate in *P. aeruginosa* or the MDR rate in *P. aeruginosa* is increasing, the carbepenem resistance rate among MDR strains has seldom been observed. The criteria for MDR *P. aeruginosa* in the study of Sekiguchi *et al.* which was resistant to imipenem, amikacin and ciprofloxacin was demonstrated 100% resistance rate to imipenem and meropenem and more than 90% resistance to arbekacin, doripenem, and aztreonam. Only resistances to polymyxin B and gentamicin were found to be significantly lower, at about 28% and 57.5%, respectively [27]. According to MDR criteria in this study which was less stringent, antibiotic resistance rates of MDR *P. aeruginosa* were higher than 50% for most antibiotics. Only doripenem susceptibility was found to be more than 60%. This lower resistance rate might be because doripenem was recently approved for use in Thailand. Interestingly, the overall resistance rate of MDR *P. aeruginosa* in Thailand was found quite high. These situations could urge an awareness of limit antibiotic usage. Only recent launched antibiotic, doripenem, was showed the resistance rate less than 40%. Thus, highly concern should be recommended especially in the strategy to prevent drug resistance emerging and to preserve sensitivity of present antibiotics.

MDR *P. aeruginosa* is a life-threatening problem that limits the use of critical antibiotics for treatment. The ratio of CR-MDR among MDR isolated was very high in hospitals, especially the university hospitals. The results of the present study indicated that those hospitals, which handled more complicated cases and thus employed more complicated antibiotic treatments, could develop more complicated drug resistance problems. Moreover, inappropiate consumption of antibiotics has been a concern in tertiary care hospitals and especially university hospitals in Thailand [3]. High amounts of drug consumption in a hospital can cause a selective pressure on microorganisms resulting in increased drug resistance [2]. This study reported significantly higher rate of CR-MDR *P. aeruginosa* in university hospitals than regional hospitals. Correlated with the study of Danchaivijitr *et al.*, the data showed that university hospitals had greater consumption of carbapenem than regional hospitals [8].

It was noteworthy that a single resistance of doripenem in CR-MDR *P. aeruginosa* was not detected in this study. This finding was correlated to lower MIC of doripenem compared to other carbapenem [28]. The doripenem single resistance could be explained by the fact that this drug was newly introduced to Thailand. This data implied that resistance mechanisms for doripenem have not yet been fully acquired by multidrug resistant strains. Moreover, double resistance of imipenem and doripenem was found in only one strain as compared to double resistance of meropenem and doripenem which could be detected in twenty-five strains. The well-known resistance mechanisms of carbapenem such as loss of porins, increasing of efflux systems and enzyme degradation, were also reported to affect doripenem [29]. Metallo-beta-lactamases were reported to affect all carbapenem, but imipenem and meropenem had different response to loss of oprD and efflux pump overexpression [29]. Increasing the efflux pumps could mainly affect both meropenem and doripenem, but not imipenem [29,30]. However, some doripenem-resistant *P. aeruginosa* clinical isolates have been found to lack functional OprD [31]. The multiple resistances of CR-MDR *P. aeruginosa* suggested the possible use of polymyxin for treatment. Although polymyxin remains active on the CD-MDR strains, polymyxin is a drug with high nephrotoxic side effect [32]. Our data suggested that treatment with doripenem might be an optional treatment instead of polymyxin to avoid side effect.

The PFGE results demonstrated a multiple DNA patterns among the resistant strains [23]. One hundred and seven clones were identified from 218 clinical isolates indicating remarkable clonal diversity. Only 4 from 107 different clones were found to be inter-hospital transmission. Two from four clones were detected in the same region and only two clones were found in different regions. Because of the difficulty in accessing patient histories, the method of transmission between hospitals could not be determined. There was a possibility that infected patients were transferred between the hospitals. The clonal dissemination ratio showed limited of inter-hospital transmission. The ratio of higher than 2.0 was found in two hospitals indicating that one clone was infected in more than two patients. Additionally, these hospitals were found dominant clones at about 9 isolates per clone as showed in the Table 2. For other six hospitals, the clonal dissemination showed that each patient was infected by different clones indicating high variation of CR-MDR *P. aeruginosa*. It was possible that high consumption of antibiotic usage could provide high pressure condition inducing mutation in bacteria.

The available results indicated that the high resistance rate of MDR *P. aeruginosa* was localized and was due to the antibiotic selection pressure. Antibiotic usage should be carefully evaluated for its effect on the development of bacterial resistance. Infectious Diseases Society of America (IDSA) has presented guidelines for developing an institutional program to enhance antibiotic stewardship [33]. The aim of the guidelines is to optimize antibiotic selection and usage while maximize the clinical outcomes. Since our results indicated that Thailand might have some patterns of resistance different from that of other countries. Therapeutic options in Thailand should be considered and adapted to minimize our resistance problems. An appropriate adjustment of antibiotic usage should reduce the emergence of antibiotic resistance microorganisms and also can preserve the existing and future antimicrobial agents [33,34].

Because CR-MDR *P. aeruginosa* infections are frequently life-threatening, strategies to control the spreading of antibiotic resistance phenomenon are necessary. Our results showed that the spreading of CR-MDR in Thailand was local, but the resistance rate of these strains was high. The high consumption of antibiotics might be a major problem. Therefore, antibiotic stewardship is one of the strategies that might help resolve the problems. The effective strategies to control the mutation of bacterial resistance are required to prevent the spreading and also antibiotic strategy for treatment.

## Competing interests

The authors declare no competing interests.

## Authors’ contributions

PK carried out the study and prepared the manuscript. PM, PM, VT and MTC supervised the work and the manuscript. MTC contributed as the principal investigator. All authors read and approved the final manuscript.
